# Photocrosslinking and photopatterning of magneto-optical nanocomposite sol–gel thin film under deep-UV irradiation

**DOI:** 10.1038/s41598-021-84376-6

**Published:** 2021-03-03

**Authors:** C. Bidaud, D. Berling, D. Jamon, E. Gamet, S. Neveu, F. Royer, O. Soppera

**Affiliations:** 1 CNRS, IS2M UMR 7361, Université de Haute-Alsace, 68100 Mulhouse, France; 2 Université de Strasbourg, Strasbourg, France; 3 Université de Lyon, CNRS, UMR 5516, Institut d’Optique Graduate School, Laboratoire Hubert Curien, Université Jean Monnet, 42023 Saint-Etienne, France; 4 CNRS, Laboratoire de Physicochimie des Electrolytes et Nanosystèmes Interfaciaux, PHENIX, Sorbonne Université, 75005 Paris, France

**Keywords:** Materials for optics, Materials science, Synthesis and processing, Optical materials

## Abstract

This paper is aimed at investigating the process of photocrosslinking under Deep-UV irradiation of nanocomposite thin films doped with cobalt ferrite magnetic nanoparticles (MNPs). This material is composed of a hybrid sol–gel matrix in which MNP can be introduced with high concentrations up to 20 vol%. Deep-UV (193 nm) is not only interesting for high-resolution patterning but we also show an efficient photopolymerization pathway even in the presence of high concentration of MNPs. In this study, we demonstrate that the photocrosslinking is based on the free radical polymerization of the methacrylate functions of the hybrid precursor. This process is initiated by Titanium-oxo clusters. The impact of the nanoparticles on the photopolymerization kinetic and photopatterning is investigated. We finally show that the photosensitive nanocomposite is suitable to obtain micropatterns with sub-micron resolution, with a simple and versatile process, which opens many opportunities for fabrication of miniaturized magneto-optical devices for photonic applications.

## Introduction

Magneto-optic (MO) effects refer to phenomena which modify the light polarization according to an external magnetic field applied to a MO active material^1–3^. One of these MO effects is the Faraday rotation (FR), which is defined as the change produced in the plane of polarization of the light transmitted through a material when a magnetic field is applied: the plane of polarization is rotated. In reflexion mode, this effect is known as the magneto-optic Kerr effect. Amongst the most attractive properties of the magnetic transparent compounds are those related to the magneto-optical (MO) effects and their scientific and industrial applications in areas such as data storage^4^, three-dimensional (3D) imaging^5^, magnonics^6,7^, sensing^8,9^ and photonics^10–12^. MO active materials are ubiquitous in photonic devices, but they are still lacking in integrated photonic platforms although they are essential components for optical communication systems (optical isolators, optical circulators, optical switches, magneto-optic (MO) modulators)^13–18^ and high performance magnetic field sensors^19–21^.

In the past, magneto-optical materials have been mainly developed by physical methods such as sputtering^22,23^, pulse laser deposition^24^, and molecular beam epitaxy^24–26^. More recently, nanocomposite approach has been proposed to simplify the synthesis of the material and its shaping into devices^27^. The principle is based on the synthesis of magnetic nanoparticles then their incorporation into a matrix which can be a polymer^28–33^, or an inorganic matrix prepared by sol–gel for example^34,35^. The materials can thus be processed by the usual processes adapted to liquid formulations. Solution-based processes appeared with the major advantage of allowing simpler processes without the need of sophisticated equipment^36^. In this context, sol–gel chemistry emerges as a very relevant and versatile solution. The principle of the sol–gel process is based on successive reactions of hydrolysis and condensation of precursors that form networks of metal oxides. One of the interests of this route lies in its compatibility with a large number of precursors. Among numerous commercially available precursors, alkoxides (alkoxysilanes zirconium, titanium, aluminium, etc.) are the most commonly used. Other derivatives are also used (chlorides, nitrates, …). The sol–gel pathway thus offers a number of advantages in terms of energy harvesting, processing versatility and a wide range of final properties^37–39^. The solutions can be deposited as thin films by simple means such as spin-coating, dip-coating or spray-coating. Moreover, the final material can reach good transparency, good mechanical properties and high refractive index. These advantages account for a wide use of sol–gel coating for optical applications.

We recently introduced a process that relies on the synthesis of a photocurable sol–gel matrix in which magnetic nanoparticles (MNP) can be introduced^40^. Cobalt ferrite (CoFe2O4) MNPs were chosen because they exhibit large Faraday rotation in the 1400–1550 nm spectral range^41^, an important criterion for potential applications in telecommunication devices and photonic integrated circuits. The major advantage of using MNPs incorporated in a non-magnetic host matrix is that the magneto-optical properties are then obtained in the final material without the need for additional thermal post-treatment. The main challenge is to manage and avert the NPs aggregation during the different steps of the process in order to preserve the magnetic properties of the individual MNPs and to avoid light scattering by aggregates in the nanocomposite. The photocurable sol–gel matrix was based on a hybrid precursor that can be crosslinked by light (namely 3-methacryloxypropyltrimethoxylsilane MAPTMS). Photocrosslinking of such matrix can be obtained by Deep-UV laser irradiation (193 nm), which opens the doors towards micro and nanopatterning. Indeed, solubility switch can be induced by laser irradiation which allows defining by laser direct writing the patterns. Other materials and patterning strategies have been proposed. In^42^, Lai et al. prepared a photopatternable material based on a commercial SU8 photoresist doped with magnetite nanoparticles and achieved high resolution patterns and 3D structures by laser direct writing.

In the present paper, we aim to investigate the photocrosslinking mechanism involved in the nanocomposite and further exemplify the application for photopatterning in the sol–gel matrix that we developed. The optimized synthesis of CoFe_2_O_4_ nanoparticles and hybrid sol–gel host matrix is first described. Conditions are defined to reach high concentration of MNPs. The kinetic of photopolymerization is then studied by Fourier transform infrared (FTIR) spectroscopy, in order to investigate the photoinduced mechanisms allowing the photocrosslinking of the material. In particular, we highlight an original photo-induced mechanism based on the excitation of Ti complexes. On this basis, a mechanism can be proposed. In a second part, DUV photopatterning is demonstrated and the effect of the composition on the final results is discussed.

## Experimental

### Synthesis of magnetic nanoparticles

The magnetic nanoparticles embedded in the matrix are cobalt ferrite nanoparticles (CoFe_2_O_4_). The preparation of these nanoparticles was performed by precipitation of cobalt chloride and ferric chloride, based on the method of Massart and Tourinho^43^. The different stages of ferrofluid synthesis can be summarized as follows: First, iron chloride (FeCl_3_) and cobalt chloride (CoCl_2_) are mixed in aqueous solution, with a molar ratio Fe/Co equal to 2. Concentrated NaOH (10 mol/L) is then added to the mixture to form the hydroxides of each metal (Fe(OH)_3_ and Co(OH)_2_). The solution is then heated to 100 °C for two hours to convert the hydroxides into cobalt ferrite. After several washing steps in water, an acid treatment is applied (overnight) by adding a 2 mol/L solution of nitric acid. This acid treatment removes hydroxides that would not have been converted into cobalt ferrite. It also makes it possible to shift from a negatively charged surface (charge due to O^-^ groups on the surface with Na^+^ counter-ions) to a positively charged surface. As cobalt ferrite particles are not stable in an acidic environment, it is necessary to protect them by a surface treatment. This treatment is carried out by adding an almost boiling solution of ferric nitrate (concentration 0.3 mol/L) to the solution containing MNP. Finally, to obtain the ferrofluid, washing steps with acetone and then ether were done and the MNP of cobalt ferrite are dispersed in water. The resulting ferrofluid is an acidic ferrofluid: the MNPs are positively charged at the surface with NO_3_^−^.

The concentration of iron and cobalt ions was determined by atomic absorption spectroscopy method after degradation of the MNPs in a highly concentrated acidic medium. The concentration was confirmed by recording the magnetization curve of the ferrofluids, measured with a vibrating-sample magnetometer (VSM, Quantum Design PPMS).

The size dispersion and morphology of the particles were determined by analysis of images obtained by transmission electron microscopy (TEM). Transmission electron microscopy (TEM) was performed using a JEOL ARM-200F microscope operating at the 200 keV accelerating voltage. The chemical analysis was performed using a JEOL Centurio detector. The samples were prepared for observation using a Leica ultramicrotome, model EM-UC7, operating at RT. To observe the film cross-section, it was deposited on the surface of a thermanox substrate. The slide thickness was ~ 100 nm.

Highest concentration in MNPs in the final materials (> 2 vol%) were reached by using a concentrated ferrofluid, obtained by dialysis. This technique consists of concentrating the ferrofluid by reverse osmosis, by immersing a porous dialysis tube filled with the ferrofluid to be concentrated in an aqueous solution of polyethylene glycol (PEG) with a mass concentration of 20,000 g/mol. Typically, dialysis of 20 mL of ferrofluid initially concentrated at 1.45 vol% for 24 h with agitation results in a highly viscous ferrofluid with an MNPs concentration of about 10 vol% (corresponding to a concentration of a factor of ~ 7). By introducing dialyzed ferrofluids into a matrix, it has been possible to achieve doping levels of up to 20 vol%, which is comparable to the highest level of MNPs in nanocomposites described in the literature^44^. The advantage of using a dialyzed ferrofluid is to introduce more magnetic nanoparticles without adding more water to the mixture.

### Sol–gel matrix and nanocomposite solution

3-methacryloxypropyltrimethoxylsilane (MAPTMS), titanium isopropoxide (TTIP), methacrylic acid (MAA), Hydrochloric acid (HCl), 1-propanol, and cyclohexanone were purchased from Sigma Aldrich. Deionized water was used throughout the reactions.

**Figure 1:**
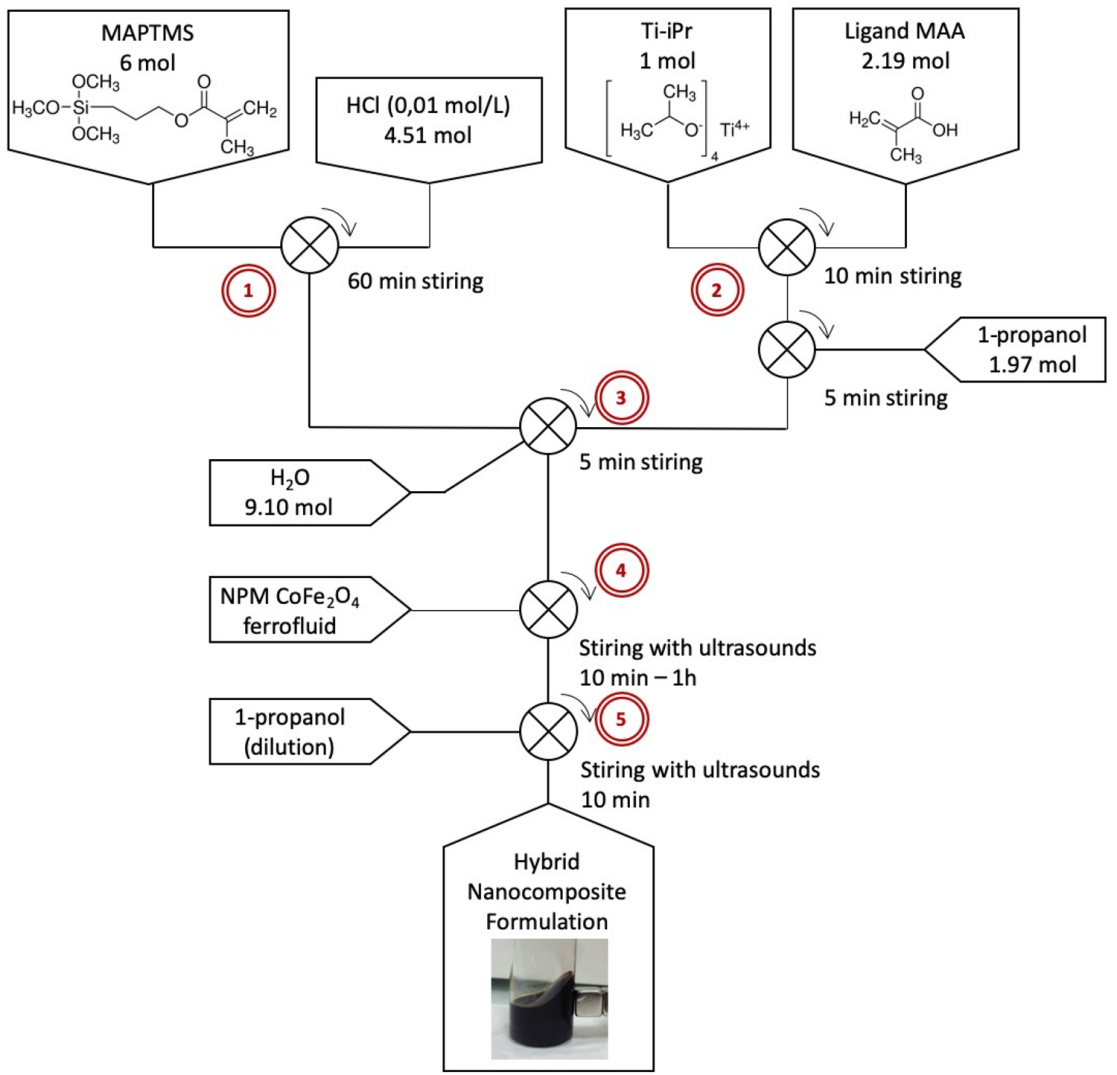
Schematic view of the preparation steps of the doped formulation, in 5 steps: 1) Pre-hydrolysis of MAPTMS, 2) Complexation of Ti precursor, 3) Hydrolysis-condensation reactions, 4) Doping of the sol–gel matrix by MNPs and 5) dilution of the formulation by 1-propanol.

The main stages of material preparation are shown in Fig. 1. It consists in 5 main steps:

1. 0.01 mol/L concentrated hydrochloric acid is added to MAPTMS (molar ratio MAPTMS/ H_2_O of 1.3:1). The solution is placed under magnetic stirring for one hour to give a colorless emulsion. The objective of this step is to pre-hydrolyze the MAPTMS^45^ and favor a homogeneous introduction of Ti precursor.
2. Methacrylic acid MAA is added to Ti precursor in a molar ratio MAA/Ti = 2.2. This molar ratio was chosen to have a large excess of MAA to ensure a full complexation of Ti precursor. Indeed, according to the literature, a minimum ratio of 1.2 is necessary for total complexation of the titanium precursor^46,47^. The mixture is magnetically stirred for five minutes. This reaction is exothermic. Subsequently, 1-propanol is added to the solution in a ratio of 0.9 molar MAA/1-propanol. The solution is homogenized with a magnetic stirrer for 10 min. A clear, bright yellow solution is obtained.
3. The solutions of MAPTMS and complexed Ti precursor are mixed together. Water is then added with a molar ratio MAPTMS/H_2_O = 0.4. This third step completes the hydrolysis-condensation steps of the sol–gel chemistry.
4. A given volume of ferrofluid containing the MNPs is added to the sol–gel solution. The doped material is homogenized by ultrasonic treatment. The stirring time varies from ten minutes to one hour depending on the amount of MNP introduced. The introduction of MNP has been considered at different stages of the process. The most stable formulations are obtained when the introduction is made at the end of the sol–gel formulation preparation steps and this process was retained (Fig. 1).
5. The doped solution is diluted with 1-propanol. The purpose of this dilution is twofold. On the one hand, the viscosity of the material must be adjusted, which will make it possible to change the thickness of the thin film during spin-coating. On the other hand, dilution ensures the stability and homogeneous dispersion of MNP in the sol. Dilution is particularly important in the case of heavily doped matrices.

The molar ratio of Si/Ti can be varied in the range 3/1 to 20/1. The reference matrix corresponds to a composition of Si/Ti in a molar ratio of 6/1. Solutions prepared are stable for several months and can be stored at room temperature.

### Thin films preparation and characterization

Substrates (Silicon wafer or glass slides) were first cleaned by rinsing with ethanol and then placed in an UV-ozone cleaner to remove the organic pollutant and increase the polarity of the substrate for good adhesion of the thin film. The formulation was filtered through 0.2 μm PTFE filters. Homogeneous films were obtained by spin coating, with typical thicknesses between 200 and 500 nm, depending on the dilution factor and rotation speed during deposition.

The photopolymerization kinetics were followed by real time-FTIR with a Thermo Scientific Nicolet 8700, coupled with a Hamamatsu high intensity mercury–xenon lamp equipped with a light guide (Lightnincure series LC8 lamp). Si wafers (thickness = 0.25 mm) were used as substrates and the irradiance was fixed to 70 mW/cm^2^. With this configuration, the sample can be irradiated in situ, which is more convenient than the ex situ laser irradiation and justifies that we used the DUV lamp for polymerization kinetics. Absorption measurements were performed with a Lambda 950 UV/Vis (Perkin Elmer).

The Faraday spectra were acquired in the wavelength range 600–1700 nm with a homemade polarimetric optical bench based on the modulation technique combined with an ellipsometric-type calibration method (see^48^ for details). The sample (nanocomposite film or ferrofluid cell) is placed perpendicular to the incident beam in the air gap of an electromagnet. The magnetic field can be varied in the ± 0.8 T range. The light from a xenon white light source combined with a monochromator passes through a polarizer, the sample, a photoelastic modulator, an analyzer, a detector, and a lock-in amplifier (LIA). This optical arrangement is suitable for analyzing the polarization state by means of the first (ellipticity) and second harmonic (rotation) signals of the LIA^49^. The calibration method^50,51^ allows to measure the absolute value of the polarization rotation with the detection limit of 0.001°.

### Photopatterning

The photopatterning setup relies on a Braggstar (Coherent) nanosecond ArF Excimer laser emitting at 193 nm^40^. The beam section measures 3 × 6 mm^2^. An attenuator located after the laser is used to tune the power. A shutter allows controlling the exposure time. A beam expander makes it possible to enlarge the beam spot by a factor of 5 and thus increases the exposed material surface. Its role is also to homogenize the beam, reduce its divergence and thus increase spatial coherence. At the exit of the expander, the beam reaches a semi-reflecting blade allowing 25% of the power to pass through and returning the remaining 75% of the power to the sample at 90°. The 25% passing through the blade allows the laser power to be measured in real time using a power meter. The sample is placed on a motorized stage in x, y and z. Displacements in x and y position the sample under the beam and the z stage adjusts the sample-interferometer distance (i.e. the focal point).

Measurements of the films thickness were done by ellipsometric spectroscopy. The measurements were performed on a UVISEL ellipsometer from Horiba–Jobin–Yvon (spectral range 190–830 nm). Data were fitted with the software from the UVISEL ellipsometer. The photopatterned films were characterized by Atomic Force Microscopy (AFM), in tapping mode, with a PicoPlus 5500 System model from Agilent.

## Results and discussion

**Figure 2:**
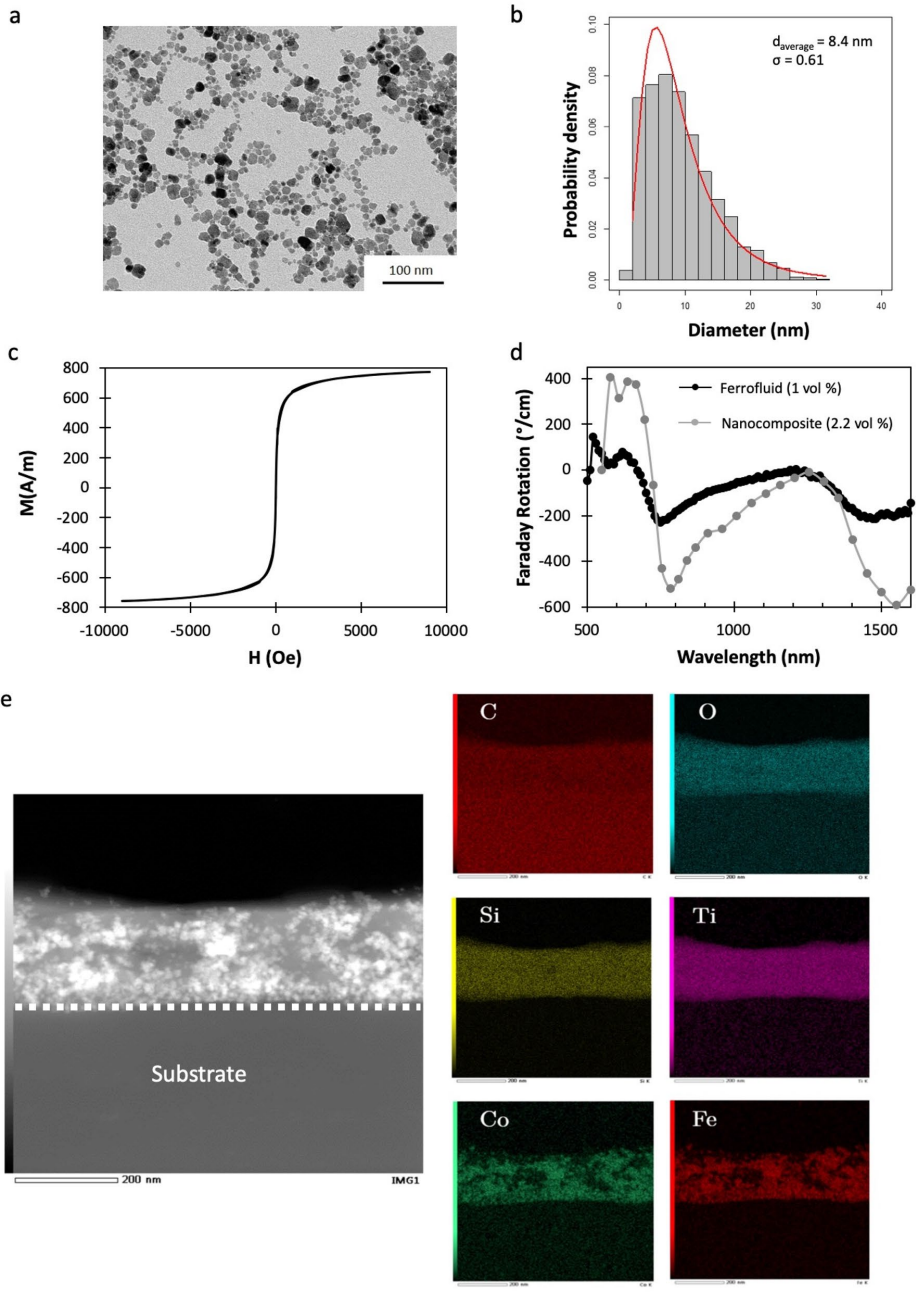
(**a**) TEM image of the CoFe_2_O_4_ NPs ferrofluid and (**b**) the probability density size distribution size obtained from TEM images. (**c**) Magnetization curve measured by VSM on the synthesized ferrofluid with a concentration of CoFe2O4 NPs of 1 vol% and (**d**) Faraday rotation spectral dependence for the ferrofluid (1 vol%) and for the sol–gel nanocomposite (molar ratio Si/Ti : 6/1, CoFe_2_O_4_ MNPs: 2.2 vol%). (**e**) STEM image and EDX analysis of a nanocomposite layer (Energies for single elements maps were chosen as follows: C:0.28 keV, O:0.52 keV, Si:1.74 keV, Ti : 4.51 keV, Co : 6.92 keV, Fe : 6.40 keV).

Structural, magnetic and magneto-optical properties of Cobalt ferrite CoFe_2_O_4_ nanoparticles were first studied. Nanoparticles were characterized by TEM. A typical TEM image is presented in Fig. 2a. The NPs size distribution fitted by a log-normal distribution gives a NPs average size of 8.4 nm with a standard deviation σ = 0.6 (Fig. 2b). The VSM hysteresis curve of the ferrofluid (given in Fig. 2c) shows the classical behavior of superparamagnetic nanoparticles. The magnetization is 800A/m and the concentration of Fe and Co ions measured by flame spectrophotometry is [Fe + Co] = 1.8 mol/L. Ferrofluids prepared under these conditions have excellent stability (several months).

Cobalt ferrite nanoparticles have been selected for their interesting magneto-optical properties. The nanocomposite material is intended for use in MO devices operating at the wavelength 1550 nm. For this reason, the spectral response of the Faraday rotation of ferrofluid has been investigated. Figure 2d shows the Faraday rotational spectral behavior of the ferrofluid synthesized according to the protocol described above. The Faraday rotation of the CoFe_2_O_4_ MNPs is particularly important around 750–850 nm, as well as in the 1500–1600 nm region, where it reaches values higher than 200°/cm. These CoFe_2_O_4_ MNPs are therefore good candidates for obtaining MO properties in these two operating windows, at especially at the 3rd telecom wavelength (1.525–1.625 nm), interesting for telecom applications.

The magneto-optical response of the thin-film material after Deep-UV curing is also shown in Fig. 2d to be compared to the response of the ferrofluid used to incorporate the MNPs. The evolution of the Faraday rotation is measured at normal incidence of a thin layer (Si/Ti: 6/1), doped to 2.2 vol% in nanoparticles. The sample thickness was 1.9 μm, deposited on a glass substrate. It was obtained by deposition of 4 layers of 480 nm, deposited and subsequently irradiated with an energy of 1.5 J/cm^2^ by the Excimer laser, which is enough to ensure that the film is dry after exposure. Crosslinking molecular phenomena will be discussed below. The thickness of MO material allows to improve the signal-to-noise ratio. As shown in Fig. 2d, the Faraday rotation of the composite material has the same spectral behavior as that of NPs ferrofluide. This result confirms that the integrity of the nanoparticles is preserved during their incorporation into the sol–gel matrix and the UV cross-linking of the thin film. The DUV cured material presents thus interesting MO properties. The structural characterization of the nanocomposite material was completed by a TEM and EDX analysis (Fig. 2e). The TEM image illustrates the good repartition of the MNPs (bright spots) within the sol–gel matrix. Si and Ti are well distributed within the film thickness, showing that the synthesis strategy is suitable to obtain an homogeneous material, which is needed for optical applications. The next sections are aimed at investigating the Deep-UV induced modification of the material leading to the crosslinking of the material and then to show how this molecular behavior can be used to direct laser write micro- and nanostructures with MO properties.

As mentioned before, we observed that the film deposited with spin-coating from the sol–gel solution doped with CoFe_2_O_4_ MNPs could be crosslinked by laser DUV irradiation. After laser irradiation, the film is tack-free and resistant to etching by organic solvents such as alcohols or cyclohexanone. As stated in the introduction, there is no study of the photoinduced phenomena in such nanocomposite materials, which is shown hereafter.

**Figure 3:**
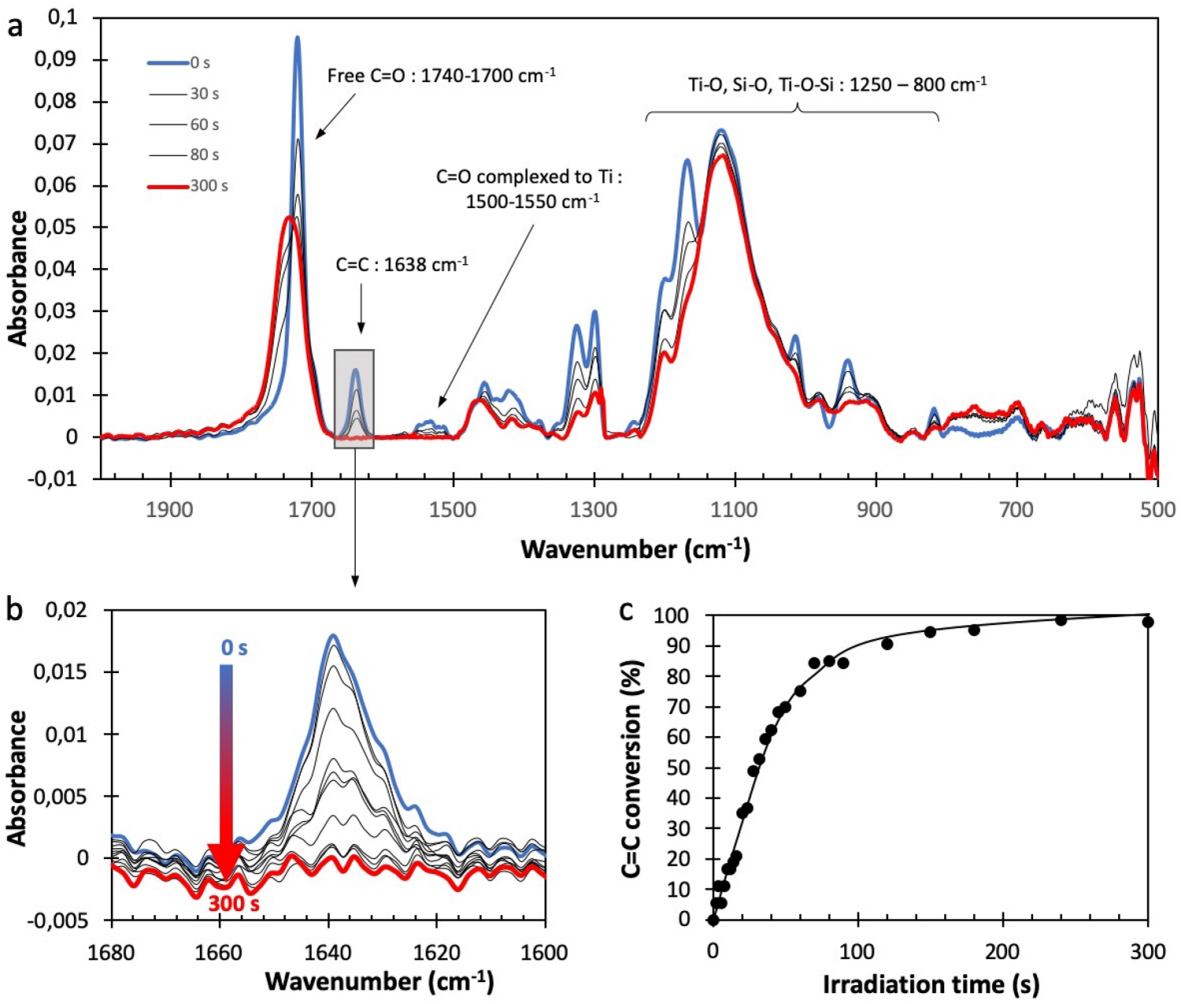
(**a**) FTIR of a nanocomposite thin film (450 nm thickness, molar ratio Si/Ti : 6/1, CoFe_2_O_4_ MNPs : 0.4 vol%) and its modification upon DUV irradiation (carried out with LC8 lamp at 70 mW/cm^2^). (**b**) Widening of the 1680–1600 cm^−1^ region to highlight the decrease of the C=C band (LC8 lamp at 70 mW/cm^2^). (**c**) Conversion of the C=C band plotted as a function of the irradiation time (line is for guiding eye).

Figure 3a displays the typical evolution of the FTIR spectrum of a nanocomposite thin film (450 nm thickness, Si/Ti = 6/1, 0.4 vol% of MNP) under DUV irradiation. Before irradiation, the absorption bands were assigned according to previous works^52^:

- The 1638 cm^−1^ band corresponds to the vibration modes of the sp2 carbons (C=C double bonds) of the material. Its presence shows that the C=C bands are not affected by the sol–gel reaction and thus available for forming the polymer network by photopolymerization.
- C=O from carboxylic acids and methacrylic functions appears at several wavenumber, according to their environment: free C=O from the methacrylate are visible at 1740–1700 cm^−1^. This position is expected for a methacrylate function, which confirms that the methacrylate can be polymerized. A second band located between 1500 and 1550 cm^−1^ corresponds to the vibrations of the C=O (methacrylic acid) complexed with titanium. This band shows the presence of the complex, formed in the early stages of the sol–gel synthesis, in the thin film after spin-coating.
- The sol–gel reaction is also confirmed by the presence of bands in the region 800–1250 cm^−1^ that can be assigned to Ti–O, Si–O and combinations thereof.

Figure 3a also shows the evolution of the FTIR spectrum during DUV irradiation. Several modifications were recorded during irradiation. The most obvious change appears at 1638 cm^−1^ (enlarged in Fig. 3b). This band gradually disappears during the irradiation, showing the consumption of the C=C double bounds. This demonstrates the polymerization of the methacrylate functions and explains the crosslinking of the material under DUV irradiation.

The conversion of the C=C can be plotted as a percentage of the C=C consumed in reference to the initial quantity of C=C bounds (Fig. 3c). The shape of the curve is classical with a maximum polymerization rate (defined as the slope of the curve) at the beginning of the irradiation and a progressive decrease of the polymerization rate with time up to a maximum conversion ratio. It confirms the very good yield of polymerization achievable with this system, despite the presence of the MNP, with a total final conversion close to 100%. The excellent final conversion is important to guarantee good mechanical and optical properties to the thin film.

Additionally, we confirmed that the decrease of the C=C double bounds (1638 cm^−1^) is not due to the loss of volatile compounds (free methacrylic acid for example). For this purpose, we followed also the evolution of the C=O bound. We observed a shift of the position of the C=O. This can be explained by the loss of the conjugation between the C=O and the C=C as the C=C is consumed by polymerization. If the area of the corresponding band is plotted versus time, the value keeps constant during polymerization, showing that there is no significant loss of material during photopolymerization.

**Figure 4:**
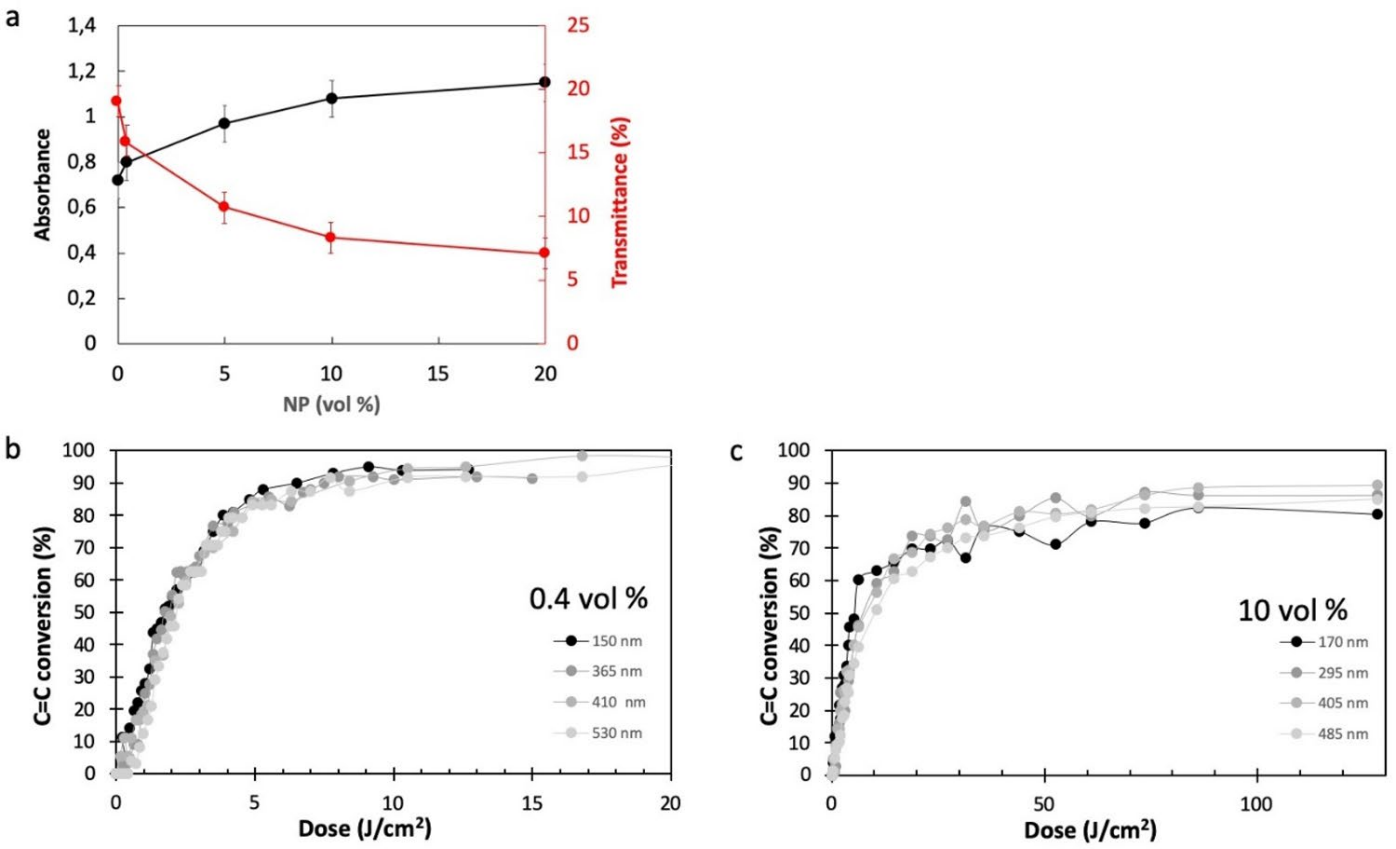
(**a**) Absorbance (black dots) and transmittance (red dots) at 210 nm of nanocomposite films (thickness: 100 nm) for different concentrations of NPs measured by UV–vis spectroscopy. (**b**) and (**c**) Conversion of the C=C band plotted as a function of the irradiation time for respectively 0.4 vol% and 10 vol% of CoFe_2_O_4_ MNPs, and for several film thicknesses in the range 150–530 nm. Irradiation was carried out with LC8 lamp (70 mW/cm^2^) and conversion was calculated from the decrease of the 1638 cm^−1^ band (molar ratio Si/Ti : 6/1).

One of the reasons explaining the excellent conversion yield of the C=C bounds in the nanocomposite is linked to the limited contribution of the MNPs to the light absorption through the film at the irradiation wavelength. Figure 4a illustrates the value of absorbance and transmittance that were determined by UV spectroscopy at a wavelength (210 nm) close to the irradiation wavelength (193 nm), for several concentrations of MNPs. The data were collected from samples with different thicknesses and the optical properties were calculated for a film thickness of 100 nm. The increase of the concentration of the MNPs only slightly increases the absorption at the irradiation wavelength, the absorption being mainly linked to the host matrix. For the highest load of MNP (20%), the contribution of the MNPs to the total absorption is about 30%. Interestingly, absorption is not linear towards MNP concentration, which can be explained by the partial aggregation of the MNPs when the concentration is increasing. The impact of the MNP on the polymerization kinetic was evaluated for several film thickness (Fig. 4b,c).

Interestingly, for the lowest MNP concentration (0.4 vol%), the photopolymerization kinetics is only slightly dependent on the film thickness and the polymerization rate and final conversion are very good for thicknesses up to 530 nm. At higher MNP concentration (10 vol%), the polymerization rate is decreased but the final conversion (80%) is high enough to insure good adhesion and mechanical properties of the thin film directly after irradiation, without any further curing. This result confirms that DUV irradiation is effective to trigger the photopolymerization of the nanocomposite. For the maximum MNP concentration achievable (20 vol%), the final conversion was 75% (for 70 J/cm^2^).

**Figure 5:**
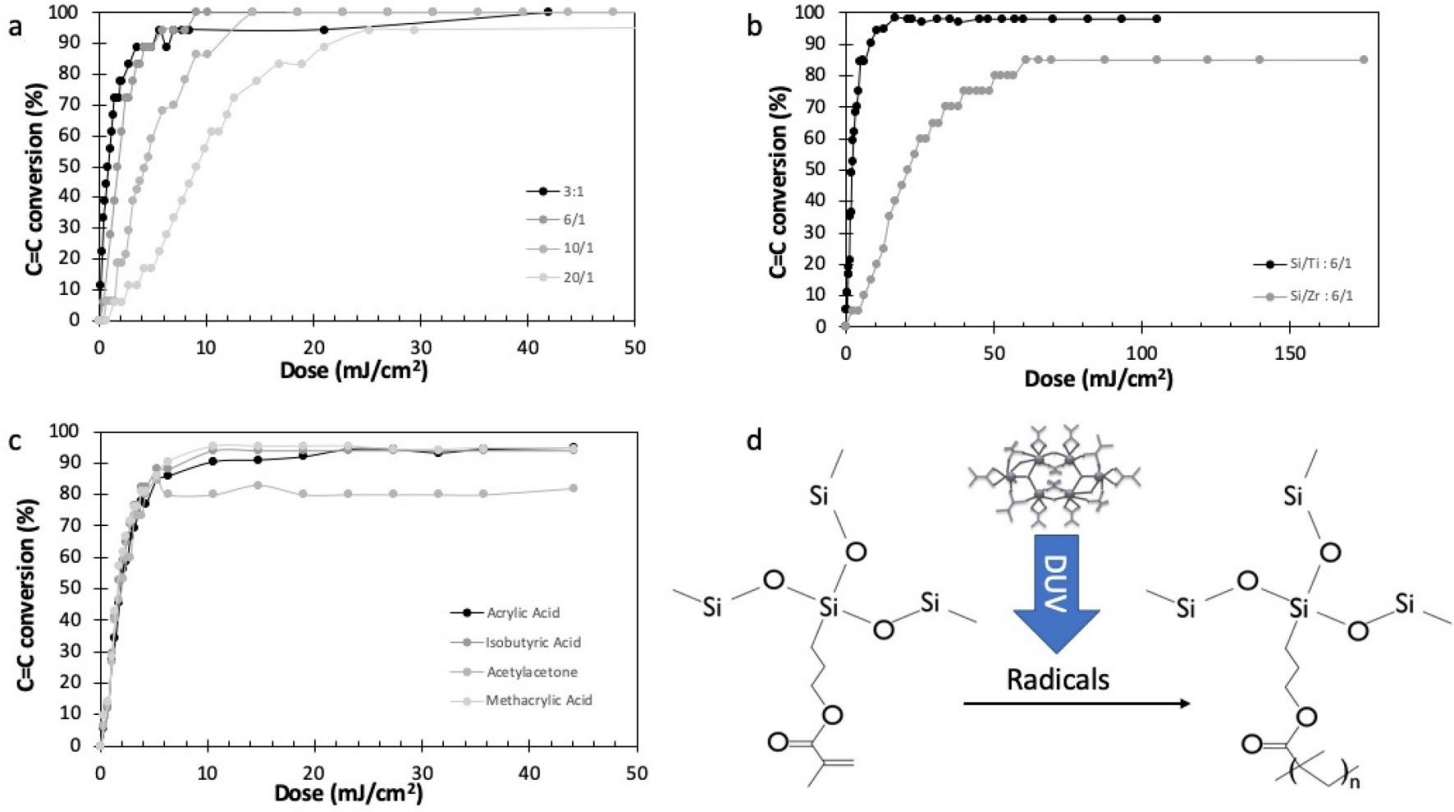
Photopolymerization of the sol–gel precursor thin film under DUV irradiation followed by FTIR (C=C conversion calculated from the decrease of the 1638 cm^−1^ band). Thickness 450 ± 20 nm, irradiation with LC8 lamp (70 mW/cm^2^), no MNP. (**a**) Influence of the Ti complex concentration on the photopolymerization kinetic (Si/Ti = 3/1 to 20/1). (**b**) Comparison of Ti and Zr complexes (Si/Zr or Ti = 6/1). (**c**) Influence of the ligands in the Ti complex (Si/Ti = 6/1). (**d**) Simplified mechanism for a free radical photopolymerization mechanism of the organic part of the hybrid sol–gel precursor.

As mentioned before, the photopolymerization process under deep-UV irradiation (193 nm) relies on the crosslinking of the organic part of the hybrid nanocomposite material. However, since there is no organic photoinitiator added in the matrix to start the polymerization, there are questions arising about the light induced mechanism accounting for the photocrosslinking. As proposed in previous studies^46,53^, metal alkoxides, when exposed to DUV light, can decompose to produce free radical species that are able to start the free radical polymerization of the hybrid matrix. We would like to confirm this mechanism for the nanocomposite material. Figure 5 displays the influence of several parameters involving the Ti complexes in order to demonstrate their central role in the photopolymerization mechanism. We first investigate the influence of the concentration of metal alkoxide on the photopolymerization kinetic (Fig. 5a).

In Fig. 5a the photopolymerization kinetic was followed by FTIR spectroscopy, with the same method as before (conversion calculated from the decrease of the C=C band at 1638 cm^−1^). This graph clearly shows that the photopolymerization is accelerated when the concentration of metal alkoxide is increased. This confirms the central role of the metal alkoxide complexed with methacrylic acid in the photopolymerization process. We observed that there is no significant improvement of the polymerization kinetics between 6/1 and 3/1 for Si/Ti ratio, which justifies the atomic ratio Si/Ti = 6/1 used in this study. Moreover, we observed that the stability of the formulation was not guaranteed after 10 days at highest load of Ti. One reason may be due to ligand exchange on the MNP surface by free methacrylic acid that is used to stabilize the metal. Also, we recorded similar kinetics after adding the MNP at low concentration (0.4 vol%), for all Si/Ti ratio, which means that there are no significant interactions between the MNP and the Ti complex acting as a photoinitiator.

Zirconium was also evaluated as a metal for photocatalyst, instead of Ti. (Fig. 5b). With Zr as a metal, the polymerization proceeds with a rate equivalent to the formulation with a very low load of Ti, which means that the photoinitiating efficiency of the Zr complex is much lower than the one of the Ti complex. Since the absorption of both Ti and Zr complexes were found to be close in the DUV range, we concluded that this difference in reactivity can be explained by a difference in redox power between the two species. The interest of Ti as a metal precursor to induce the free radical polymerization of the hybrid sol–gel is thus demonstrated.

Finally, the nature of the ligand used to complex the titanium alkoxide precursor was also investigated. Figure 5c presents the FTIR kinetic study of the polymerization for different ligands in Ti complexes. Four different ligands were used to complex TTIP: three carboxylic acids (acrylic acid, isobutyric acid and methacrylic acid) and a β-diketone (acetylacetone). In each case, the ligand/Ti ratio was maintained at 2.2 molar % (without MNPs). No significant difference in polymerization kinetics was observed for the different complexing agent, with comparable polymerization rate and final conversion. The ligand chosen to complex the titanium therefore has little influence on the polymerization of the composite formulation. In Fig. 5d, we propose a simplified mechanism to summarize the role of the Ti-complexes in the elaboration of the nanocomposite material. It is admitted that the chosen experimental conditions lead to the incorporation of the Ti complexes as Ti-oxo clusters, as schematized in Fig. 5d.^42^ The decrease of the band located between 1500 and 1550 cm^−1^ that corresponds to the vibrations of the C=O (methacrylic acid) complexed with titanium demonstrates the photolysis of the Ti-oxo clusters under DUV. This result is relevant with the results proposed in previous study, for comparable cluster, but in different conditions. The reactive species are able to trigger the free polymerization of the acrylate functions. The very good polymerization yield suggests a free radical mechanism. Note that the free radical formation of such clusters under DUV irradiation was already proposed in previous studies^46^. Such a property of the Ti complexes suggests that there is no interest to add any organic photoinitiator into the formulation to improve the photopolymerization efficiency. Several commercial photoinitiator known for their efficiency in free radical photopolymerization were added with a concentration of 2 wt% (Irgacure 184, 369 et 819 from Ciba). The corresponding polymerization kinetics revealed only a slight increase of the polymerization rate. This improvement is minor and thus the addition of an organic photoinitiator inside the formulation is not justified.

**Figure 6:**
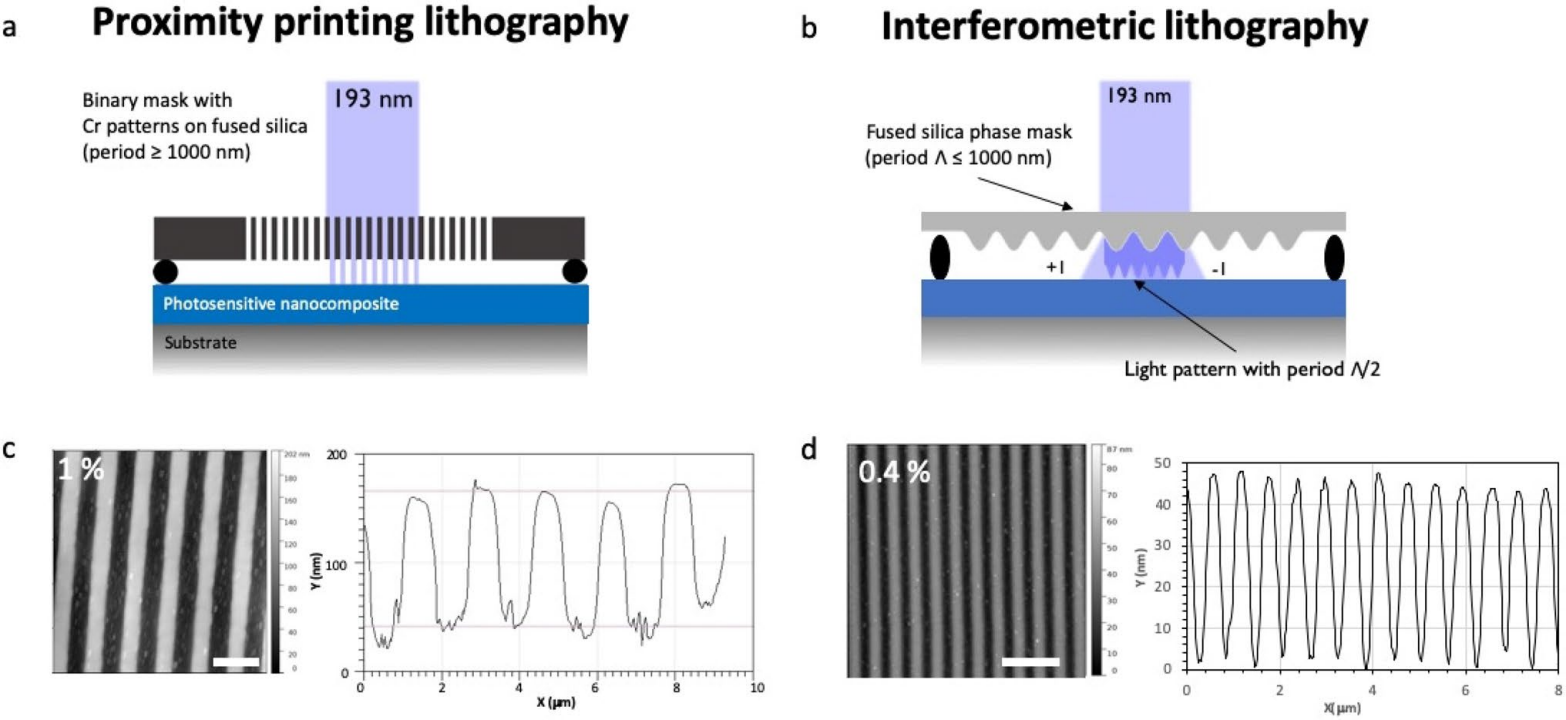
Schematics of the photopatterning setups used in this study: (**a**) proximity printing and (**b**) interferometric lithography. AFM images and cross-sections of typical examples of structures obtained in (**c**) proximity printing (period: 1600 nm, width of structures: 800 nm, height: 125 nm, MNP: 1 vol%) and (**d**) interferometric lithography (period: 600 nm, width of structures: 300 nm, height: 50 nm, MNP: 0.4 vol%). Scale bars are 2 μm in each AFM images.

In this final part, we discuss the potential of this formulation to be used as a negative tone photoresist to produce sub-micrometric patterns at room temperature with magneto-optical properties. Indeed, as shown before, a very efficient Photocrosslinking can be obtained by DUV irradiation, which can be used for photopatterning the MO material. For this purpose, two home-made photolithography setups were used, as depicted in Fig. 6, in order to show the performance and versatility of this material for photolithography applications: for the lowest resolutions (typical feature lateral size superior to 1000 nm), a proximity printing setup was used, consisting in binary masks (chromium patterned deposited on fused silica substrates) placed close to the sol–gel film (Fig. 6a). In this configuration, the metal lines cut the DUV lights and prevent the Photocrosslinking reaction to occur. For highest resolutions, in order to overcome diffraction problems, an interferometric lithography setup was used (Fig. 6b). With this setup, the period of the patterns can be varied accordingly to the phase mask used. In the present study, we focused on patterns with period of 500 nm generated by a phase mask having a period of 1000 nm. The interference approach allows higher resolutions but, in this case, the light pattern is sinusoidal, since the contrast is generated by the interference between the two diffracted beams. In this configuration, there is thus no 0-light area, which can have some consequences on the shape of the structures, as shown later.

The coatings were prepared by spin-coating on a substrate cleaned with UV-ozone cleaner. The spin-coating rotation speed and dilution factor of the solution were adjusted to obtain the desired thickness. After irradiation with one of the two configurations described above, the sample is directly developed in a solvent to dissolve the non-irradiated parts. The material behaves like a negative resin, with irradiated parts becoming insoluble. The nature of the solvent as well as the development time have been optimized. Water cannot dissolve the unexposed parts thus cannot be used as a developer for this material. Alcohols (ethanol, methanol) and acetic acid are too strong developers and dissolve the exposed parts and are thus not suitable neither. Cyclohexanone was proved to constitute a good candidate and was chosen in the following as a solvent for development. Well-defined structures could be obtained after 10 s. development in cyclohexanone. No thermal annealing is required after development for stabilization of the sample as a post-treatment. We observed that the time between sample preparation and irradiation shall not exceed 10 min, otherwise, the development is more difficult to carry out. This is due to the condensation reaction that can occur at room temperature, because of atmosphere moisture. This condition is not limiting since the typical irradiation times are shorter than this value (few sec. to few tens of sec.).

Figure 6c,d show typical patterns obtained in both photopatterning configurations. In both cases, photopatterning could be demonstrated. For the proximity printing lithography (period 1600 nm with line width of 800 nm), well-defined patterned with free substrate between lines and low line edge roughness could be demonstrated (Fig. 6c). The patterns height was 125 nm for a deposited film thickness of 150 nm. We attributed the loss of height to the shrinkage occurring in the material upon DUV irradiation (partial loss of organic moieties). These results illustrate that patterns with width of 1000 nm or more can be obtained.

**Figure 7:**
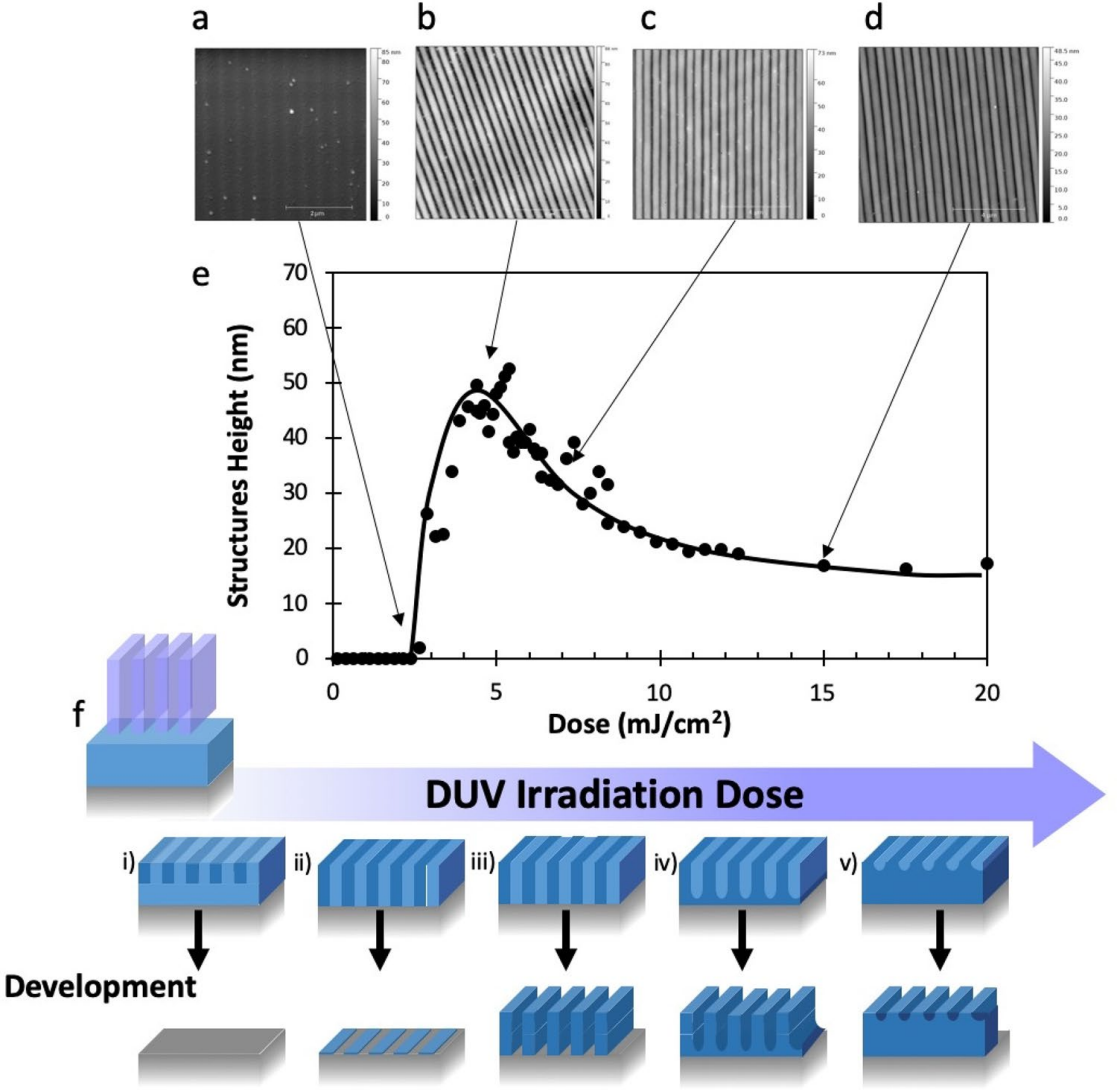
AFM images of patterns written with various doses in interferometric configuration: (**a**) 2.5 mJ/cm^2^, (**b**) 5 mJ/cm^2^, (**c**) 7.5 mJ/cm^2^, (**d**) 15 mJ/cm^2^. Period 500 nm. (**e**) Variation of the pattern height with the dose. (**f**) Schematic interpretation of the evolution of the pattern structure with the irradiation dose. Initial film thickness 80 nm. The solution has a molar ratio Si/Ti = 6/1 and the MNPs concentration was 0.4 vol%.

In interference lithography, patterns were obtained with higher resolutions but as shown in example in Fig. 6d, the pattern height was lower. Indeed, in this example, though the film thickness was decreased to 80 nm, patterns height was only 50 nm. This result was interpreted as a residual layer remaining between written lines. In order to further investigate the behavior of the material in these conditions, a systematic study of the DUV photopatterning was conducted. Results are exposed in Fig. 7.

The period of the patterns in Fig. 7 was 500 nm (corresponding to a line width of 250 nm with a space of 250 nm). The heights of the structures were measured by AFM. They are plotted in Fig. 7e. Figure 7a–e show the AFM images of surfaces irradiated with respectively 2.5, 5, 7.5 and 15 mJ/cm^2^. Figure 7f gives a schematic interpretation of the evolution of the pattern structure with the irradiation dose. For the lowest doses (less than 2.5 mJ/cm^2^), no pattern was observed. We explain this response by a too low conversion within the irradiated parts and thus the crosslinking of the matrix is not enough to promote the adhesion of the material on the substrate during development (case **i)** in Fig. 7f). This behavior corresponds to a too low conversion of the C=C bond in the organic part of the hybrid matrix, especially at the resin-substrate interface due to the internal filter effect. At 2.5 mJ/cm^2^, the conversion of the organic matrix is sufficient to give rise to a very thin layer of crosslinked material at the substrate surface. It explains why in Fig. 7a, patterns are observable but with a height much smaller (a few nm) than the initial film thickness (80 nm). From 2.5 mJ/cm^2^ to 5 mJ/cm^2^, the measured height increases rapidly with the dose as crosslinking proceeds more and more efficiently in the photoresist. However, the case depicted in Fig. 7f-iii is never reached since the maximum height (50 nm, Fig. 7b) was always significantly lower than the initial film thickness (80 nm). This explains the apparition of a residual layer between lines (Fig. 7f-iv) due to the irradiation in dark fringes of the interference pattern. This assumption is confirmed by the gradual decrease of the pattern height with dose (Fig. 7c,d) that corresponds to the increase of the thickness of the layers between lines. Such behavior is partially explained by the interference pattern irradiation configuration. Indeed, one of the drawbacks of this configuration is that the light intensity is sinusoidal so it is not null in the dark fringes.

**Figure 8:**
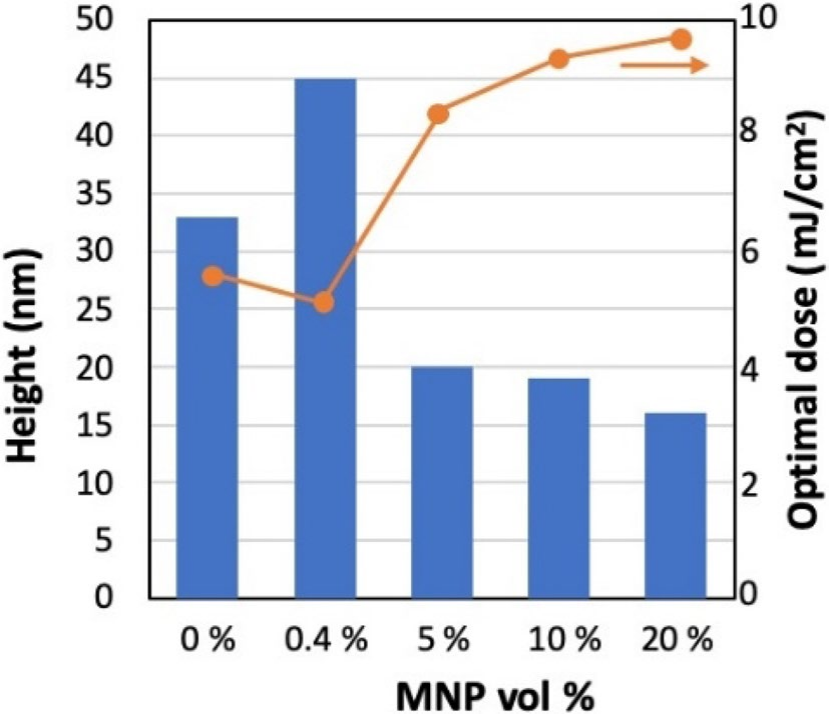
Structure height and optimal dose to obtain this height as a function of the MNP concentration. Interference lithography, Period: 600 nm.

As shown in a previous study^40^, the concentration of the MNP has an impact on the photopatterning. Patterns with various concentrations of MNP (between 0 and 20 vol%) were prepared using interference lithography (period 600 nm). Photopatterning can be obtained in this wide range of MNP concentration but there is a strong impact of the MNP load on the quality of the patterns and their height. In particular for the highest loads of MNPs we observed the apparition of roughness at the sample surface after development that can be linked to partial aggregation of the MNPS that occurs at the surface of the patterns. These aggregated nanoparticles may create bridges between close structures, which may account for the difficulty to conduct development in these conditions, which results in remaining material between lines. In Fig. 8, we plotted the typical maximal height and optimal dose for the different MNP concentrations. Interestingly, Fig. 8 reveals that the optimal dose is increased, as expected, with the content of MNP, but only slightly in a 80 nm thin film. In conclusion, photopatterning with submicron resolution is achievable with MNP concentrations as high as 20 vol% but in this case, a significant decrease of the pattern modulation, due to the presence of a residual layer between written lines is observed. However, for many applications as gratings or in guided optics, such residual layer is not a problem for practical applications since it can be taken into account in the design of the optical design to produce a given optical function.

**Figure 9:**
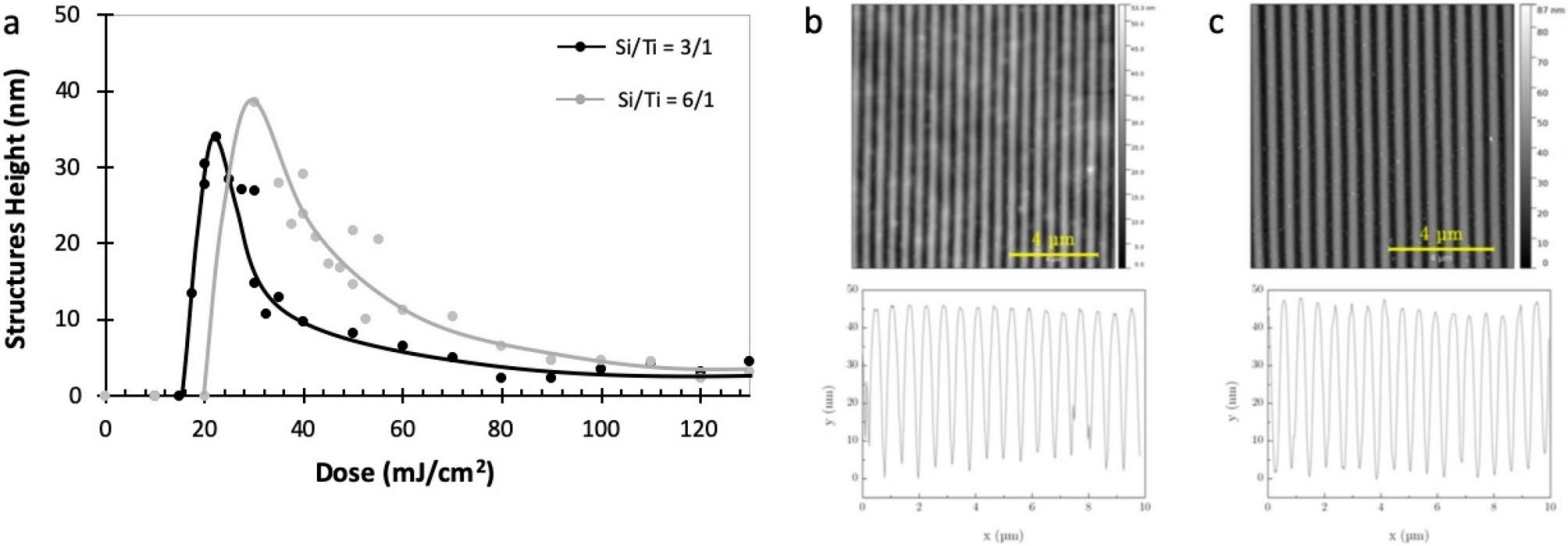
(**a**) Variation of the pattern height with the dose for two content of Ti complex in the material: Si/Ti = 3/1 and 6/1 molar. Initial film thickness was 80 nm, period 500 nm, MNP: 0.4 vol%. (**b**) and (**c**) are AFM images of the patterns for the optimal dose, respectively for Si/Ti = 3/1 and 6/1.

Finally, in Fig. 9, we show the impact of the Ti complex concentration to confirm that the patterning is indeed triggered by the Ti complex, as suggested by the polymerization kinetic studies shown previously, and to evaluate the impact of the Ti complex concentration on the patterns.

Two concentrations of Ti complexes were used (Si/Ti = 3/1 and 6/1), with the same concentration of MNP (0.4 vol%). In both cases, a residual layer between lines is obtained after photolithography. As expected, the dose needed to achieve the photopatterning of the material is lower for the higher content of Ti, which confirms the role of Ti complex as a photoinitiator of the crosslinking reaction within the material. However, the maximum pattern height was obtained for the lower Ti content, which confirms the interest to use a molar ratio Si/Ti = 6/1, as mentioned previously. Increasing the content of Ti allows decreasing the exposure time for a given power but finally, no significant improvement of the maximum height was observed.

In conclusion, we have shown in this paper that DUV photolithography (193 nm) is an extremely interesting tool for the micro and nanostructuring of thin films with magneto-optical properties. Starting from solutions whose composition can be easily adapted to modulate the properties, the DUV photolithography step allows to cross-link the material and to structure it at submicrometer scales. No additional step (in particular no thermal annealing) is required to obtain the magneto-optical properties, which opens perspectives for the integration of these materials in devices, on glass, on silicon, but also on plastic.
